# PBMCs Mitochondrial Respiration and Its Relation to Immunity, Fitness, and Metabolic Risk in the Healthy Elderly

**DOI:** 10.1002/jcp.70096

**Published:** 2025-09-27

**Authors:** Kristina Gebhardt, Anne Hebecker, Natascha Sommer, Robert Ringseis, Klaus Eder, Magdalena Huber, Hartmann Raifer, Karsten Krüger, Christopher Weyh

**Affiliations:** ^1^ Department of Exercise Physiology and Sports Therapy, Institute of Sports Science Justus‐Liebig‐University Giessen Germany; ^2^ Department of Internal Medicine Universities of Giessen and Marburg Lung Center (UGMLC), Member of the German Center for Lung Research (DZL), Excellence Cluster Cardio‐Pulmonary Institute (CPI), Justus‐Liebig University Giessen Germany; ^3^ Institute of Animal Nutrition and Nutrition Physiology Justus Liebig University Giessen Giessen Germany; ^4^ Center for Sustainable Food Systems Justus Liebig University Giessen Senkenbergstraße 3 Giessen Germany; ^5^ Institute for Systems Immunology Center for Tumor and Immunology Marburg Germany

**Keywords:** aging, cell respiration, immune system, inflammation, mitochondria, peripheral blood mononuclear cells, physical fitness

## Abstract

Mitochondrial function plays a central role in regulating immunological and metabolic processes, particularly during successful aging. This cross‐sectional study aimed to investigate associations between mitochondrial respiration of peripheral blood mononuclear cells (PBMCs; MR_PBMC_) and key markers of immune function, systemic inflammation, and metabolic health in a cohort of healthy older adults. Sixteen healthy, physically active participants aged > 55 years (male: *n* = 9; female: *n* = 7; age: 64 ± 3.7 years; BMI: 24.3 ± 2.9; VO_2peak_: 31.1 ± 8.8 mL/min/kg) were recruited. Participants were tested for their maximal oxygen uptake (VO_2peak_) as well as cardiovascular and metabolic risk factors. Venous fasting blood samples were collected. For further analysis, MR_PBMC_ was measured using the Oroboros O2k‐Oxygraph. T cell subsets were analyzed by flow cytometry, serum cytokines by LUMINEX assays, and gene expression by qPCR analysis. We found positive associations between basal and maximal MR_PBMC,_ and the percentage of CD4^+^ T cells, with a notable link to naïve CD4^+^ T cells (*p* < 0.05). Maximal MR_PBMC_ was negatively associated with proportion of effector memory CD4^+^ T cells (*p* < 0.05). Basal MR_PBMC_ showed negative associations with pro‐inflammatory serum cytokine tumor necrosis factor alpha (TNF‐α), while maximal MR_PBMC_ was positively associated with interleukin 8 (IL‐8), intercellular adhesion molecule 1 (ICAM‐1), and vascular endothelial growth factor (VEGF) (*p* < 0.05). Intracellular signaling markers, including mRNA level of signal transducer and activator of transcription 3 (STAT3), also showed positive associations with maximal MR_PBMC_ (*p* < 0.05). No correlations were found for variables such as cardiorespiratory fitness, IL‐6, and IL‐10. In conclusion, PBMC mitochondrial bioenergetics are linked to T cell subpopulations and systemic inflammation in healthy older adults. Higher mitochondrial respiration reflecting better mitochondrial function favors a more naïve CD4^+^ T cell distribution. In contrast, lower mitochondrial function was observed in individuals with a more pro‐inflammatory profile, suggesting a potential relationship between immune status and mitochondrial bioenergetics in older adults.

## 1 Introduction

The physiology of aging encompasses a complex array of biological processes that contribute to the gradual decline in functional capacity observed in living organisms over time (J. Guo et al. 2022; Y. Li et al. 2024). Aging is marked by significant metabolic changes, including alterations in overall energy production, nutrient sensing, and cellular mechanisms. These metabolic shifts are often associated with increased systemic inflammation and weakened immunity (Chen et al. 2022; Yang et al. 2024). Central to these processes are mitochondria, whose dysfunction is increasingly recognized as a pivotal factor in metabolic and immune aging (Harrington et al. 2023; Memme et al. 2021). Due to their role in energy metabolism, mitochondria are important modulators of immune responses. For example, activation of T cells involves increased glucose and glutamine oxidation, and in particular glucose oxidation, while oxidative phosphorylation (OXPHOS) remains necessary for functional differentiation (Almeida et al. 2016; Conte et al. 2020; O'Neill et al. 2016). After their activation, immune cells switch back to OXPHOS, for which integrated and functionally stable mitochondria are important to restore the immune balance (O'Neill et al. 2016). Accordingly, mitochondrial respiration, particularly basal and maximal respiration, serves as a critical indicator of mitochondrial function and cellular energy production, which are directly linked to an individual's physiological performance and ability to adapt to physical stress. These measures reflect the cell's capacity to generate energy efficiently under varying conditions, making them essential for understanding overall metabolic health (Avram et al. 2022; Divakaruni and Jastroch 2022; Yu and Pekkurnaz 2018).

In senescent cells, mitochondria undergo structural changes that are often associated with increases in their size and volume, primarily due to the accumulation of dysfunctional mitochondria from alterations in the mitochondria‐specific autophagy (‘mitophagy’) degradation process. These age‐related changes in mitochondrial turnover as well as a NAD^+^/NADH imbalance, mitochondrial Ca^2+^ overload, mtDNA mutations, and altered nutrient signaling can cause mitochondrial dysfunction, which can be defined as a decreased respiratory capacity per mitochondrion and decreased mitochondrial membrane potential (Miwa et al. 2022). These processes are followed by an impaired energy metabolism, which is related to changes in the six main metabolic pathways that regulate metabolism in immune cells: glycolysis, tricarboxylic acid (TCA) cycle, amino acid metabolism, pentose phosphate pathway, fatty acid oxidation (FAO), and fatty acid synthesis (Conte et al. 2020; O'Neill et al. 2016).

Not only age, but also various inflammatory diseases seem to impair the metabolic capacity of mitochondria in immune cells often reflected in an altered immune cell respiration. Recent studies showed these impaired mitochondrial bioenergetics of various immune cells in different chronic and inflammatory diseases compared to healthy controls (Krüger and Gebhardt 2024). PBMCs represent mononuclear immune cells composed of monocytes, dendritic cells, and lymphocytes (including T, B, and NK cells). In PBMCs, Sommer et al. (2022) found a negative correlation between mitochondrial respiration and disease severity in patients with pulmonary arterial hypertension. Similarly, P. Li et al. (2015) detected lower maximal respiration and negative correlations between mitochondrial respiratory function and inflammatory cytokines and cardio‐metabolic risk factors.

In contrast to the negative effects of aging, a healthy lifestyle seems to have a positive impact on the development of chronic and inflammatory diseases. In particular, a nutritious diet, regular physical activity, and maintaining good mental health can significantly delay the aging process (X. Li et al. 2023). Tyrrell et al. (2015) revealed associations between PBMC bioenergetics with key physical markers of aging, reduced physical function, muscle strength and quality, and higher plasma IL‐6 levels in obese older adults. While there is evidence that long‐term training interventions, such as endurance exercise or high‐intensity interval training, are suitable for improving mitochondrial respiration of immune cells (Krüger and Gebhardt 2024), other studies have shown improvements in skeletal muscle mitochondria bioenergetics (Menshikova et al. 2006; Tonkonogi et al. 2000). However, in healthy individuals, PBMC mitochondrial function does not necessarily reflect skeletal muscle mitochondrial function (Hedges et al. 2019). Yet, in clinical populations such as patients with mitochondrial disorders, PBMCs may serve as a surrogate marker of skeletal muscle mitochondrial dysfunction (Alonso et al. 2021).

Therefore, the present study aimed to explore the associations between mitochondrial respiration in peripheral blood mononuclear cells (PBMCs) and key markers of immune function, systemic inflammation, and metabolic health in a cohort of healthy older adults. Recognizing the central role of mitochondrial bioenergetics in regulating immunometabolic processes during aging, we specifically sought to examine how basal and maximal mitochondrial respiratory capacity relate to the distribution of T cell subpopulations, circulating cytokine levels, and intracellular signaling pathways. Furthermore, we investigated whether mitochondrial respiration correlates with cardiorespiratory fitness and metabolic risk factors, including glucose metabolism and body composition. By focusing on a particularly healthy, medication‐free, and non‐diseased cohort, we aimed to identify physiological variation in mitochondrial and immune parameters that may be obscured in clinical populations. Given the exploratory nature of this study, we applied a cross‐sectional study design to capture interindividual differences under steady‐state conditions. This approach was chosen to generate hypotheses regarding potential biological interactions that may inform future longitudinal or interventional studies targeting mitochondrial resilience and immune function in aging populations.

## 2 Materials & Methods

### 2.1 Study Design and Participants

In this cross‐sectional study, 16 participants (9 males; 7 females, age: 64 ± 3.7 years; BMI: 24.26 ± 2.9 kg/m²) were included. These participants form a subgroup of the Giessen Immune aging Study cohort, which has been thoroughly described in a previous publication, including details on baseline characteristics and endurance performance (Böttrich et al. 2024). All participants were in good health and free from any acute illnesses, including infections or injuries. Recruitment for this study occurred between August 2020 and December 2021. Inclusion criteria required participants to be over 55 years old, with women being postmenopausal. Exclusion criteria included excessive alcohol consumption ( > 2 drinks/day), smoking, BMI < 18.5 kg/m², history of cardiovascular (e.g., myocardial infarction), neurological (central/peripheral), obstructive pulmonary, or metabolic diseases (diabetes type 1/2), as well as systemic illnesses (e.g., cancer, arthritis, hepatitis, HIV, autoimmune diseases). Participants were also excluded if they had used immunomodulatory medications within the past 12 weeks or received treatment for specific diseases. The medical ethics committee of the Justus‐Liebig‐University Giessen approved this study (AZ 100/20). All experimental procedures were performed according to the Declaration of Helsinki, and all participants gave written informed consent before enrolment. Participants were instructed to refrain from exercising the day before the appointment. In case of acute illness (e.g. respiratory infection), the appointment was postponed until full recovery. Fasting venous blood samples were collected between 08:00 and 10:00 am. A volume of 7.5 mL whole blood was drawn into serum gel CAT monovettes and 7.5 mL into K3E‐EDTA tubes. Serum was separated and stored at −80°C for cytokine analysis, while anticoagulated whole blood was processed for mononuclear cell isolation. For flow cytometry, 7.5 mL lithium‐heparin (LH) tubes were used. Body composition (FFM%) was assessed via bioelectrical impedance analysis (BIACORPUS RX 4004 M, MEDI CAL HealthCare GmbH). Cardiopulmonary exercise testing was performed on an electrically braked cycle ergometer (Excalibur Sport®, Lode) using individual ramp protocols to reach maximum load within 15 min. Maximum voluntary grip strength of the dominant hand was assessed using a hand dynamometer (Baseline® Hydraulic Hand Dynamometer LiTE®, Fabrication Enterprises Inc., US). Anthropometric data are summarized in Table 1.

**Table 1:** Anthropometric data shown in mean ± SD with maximum, minimum, and range; *n* = 16 participants (male = 9) were used.

	Mean + SD	Maximum	Minimum	Range
Age	64 ± 3.72	73	59	14
BMI	24.26 ± 2.89	30.57	19.26	11.32
FFM (%)	73.31 ± 6.64	86.09	65.33	20.76
VO_2peak_	31.06 ± 8.83	49	23	26
Male:	36.00 ± 8.87	27	49	22
Female:	24.71 ± 2.50	30	23	7

Abbreviations: BMI, body‐mass‐index; FFM, fat‐free body mass; VO_2peak_, maximal oxygen uptake.

### 2.2 Isolation of Peripheral Blood Mononuclear Cells

To isolate PBMCs, fresh peripheral blood was mixed with an equal volume of phosphate‐buffered saline (PBS) and placed on top of EasySep in SepMate 50 mL tubes. After centrifugation at 1200 × g for 10 min, the upper layer was carefully discarded. The isolated cells were then washed and centrifuged again at 300 × g for 8 min. Subsequently, the PBMCs were resuspended in Bambanker® freezing medium containing 10% dimethyl sulfoxide (DMSO) and frozen. The cells were stored at −80°C for later analysis. Samples were collected between December 2021 and March 2022 and stored for approximately 6 months until measurement.

### 2.3 High Resolution Respirometry of PBMCs

Frozen PBMCs were thawed at 37°C, washed twice in RMPI 1640 (Gibco, UK) containing 10% FBS (Gibco, UK), then pelleted, and resuspended in mitochondrial respiration media (89 mL RPMI, 1 mL Penicillin‐Streptomycin + 10 mL Hepes (0.2% of Hepes Stock Solution). After thawing, PBMC viability was assessed using the CASY Cell Counter and Analyzer System (OLS OMNI Life Science, Germany). This system determines cell viability based on electrical current exclusion, providing a reliable and label‐free measure of viable versus nonviable cells. Only samples with a viability ≥ 85% were used for subsequent high‐resolution respirometry. Afterward, mitochondrial respiration of PBMCs was determined using an Oroboros‐2‐k oxygraph (Oroboros Instruments, Innsbruck, Austria) for high‐resolution‐respirometry (HRR) by using a substrate‐uncoupler‐inhibitor titration (SUIT) protocol. For each participant, experiments were performed once as singlets at 37°C in a 0.5 mL chamber filled with 4 to 6 ×10^6^ PBMCs. Air and background calibrations were performed as recommended. First, the initial respiratory rate (basal respiration) was measured. For quality control, we applied oligomycin (2 μL of 1,25 mM oligomycin) to inhibit ATP synthase and measure LEAK respiration. To assess mitochondrial coupling efficiency, the ratios Leak/ETS (L/E) and (ETS−Leak)/ETS (E − L/E) were calculated. The mean value for L/E was 0.310 (SD  =  0.14; 95% CI: 0.23–0.38), and for E − L/E 0.69 (SD  =  0.14; 95% CI: 0.61–0.76), indicating preserved mitochondrial coupling and intact membrane integrity in PBMCs. Afterward, stepwise titration of 2 μL of 1 mM carbonyl cyanide p‐trifluoro‐methoxyphenyl hydrazone (FCCP) was conducted to measure the maximal (uncoupler‐stimulated) mitochondrial respiratory capacity. Last, 4 μL of 5 mM antimycin A was titrated to shut down complex III of the ETC. Data were recorded in DatLab version 4.3 (Oroboros Instruments, Innsbruck, Austria). A typical primary recorded experiment is shown in the Supporting Material Figure S1.

### 2.4 T Cell Phenotyping by Flow Cytometry

To analyze T cells and their subsets, frozen PBMCs were thawed at 37°C, washed twice in RPMI 1640 (Gibco, UK) containing 10% FBS (Gibco, UK), and then pelleted. The cells were resuspended in PBS at a concentration of 1 × 10⁶ cells/mL. The cells were incubated in the dark at room temperature for 20 min with an optimized amount of each antibody (CD3 (FITC), CD4 (AF700), CD25 (APC), CD45RO (PerCP/Cyanine5.5), CD197/CCR7 (BV421), and CD127 (PE)). For the gating strategy to identify regulatory T cells (T_regs_) and their subsets, lymphocytes were first selected based on forward scatter/side scatter (FSC/SSC) in a dot plot, with a minimum of 250,000 events per tube. Singlets were identified within the lymphocyte gate. A live/dead stain was performed using Zombie Aqua™ to identify viable lymphocytes (BioLegend Inc., San Diego, CA). Among these live cells, the frequency of CD3^+^CD4^+^ cells was evaluated. T effector cells (T_eff_) were gated as CD4^+^CD25^+^CD127_high_, while T_regs_ were defined as CD4^+^CD25^+^CD127_low_. T_reg_ subsets were further gated as resting‐T_regs_ (rT_regs_) (CD45RO^‐^CCR7^+^) and memory‐T_regs_ (mT_regs_) (CD45RO^+^CCR7^‐^). The ratio of T_regs_ to T_eff_ was calculated by dividing the total number of T_regs_ by the total number of T_eff_ cells. Flow cytometry analysis was performed using a CytoFLEX S (Beckman Coulter) and Kaluza analysis software 2.1 (Beckman Coulter), with compensation applied for spectral overlap when using multiple colors. For the immune aging panel, PBMCs were thawed, washed, and rested in pre‐warmed RPMI/5% AB serum for 2 h. The staining for the second panel (Immune‐aging panel) was performed immediately after the resting period using the Zombie NIR fixable viability kit, CD4 (AF700), CD8 (BV510), CD27 (PE), CD28 (BV421), CCR7 (PE/Cy7), CD45RA (PerCP), CD95 (FITC), and PD1 (APC), all from BioLegend (BioLegend Inc., San Diego, CA). For the immune aging panel, CD4^+^ and CD8^+^ T cells were gated after identifying live cells using the Zombie NIR fixable viability kit. T cells were then classified into subsets based on their CCR7 and CD45RA expression: naïve T cells (CD45RA^+^CCR7^+^), effector memory (EM) T cells (CD45RA^‐^CCR7^‐^), central memory (CM) T cells (CD45RA^‐^CCR7^+^), and effector memory cells re‐expressing CD45RA (TEMRA) T cells (CD45RA^+^CCR7^‐^).

### 2.5 Determination of Serum Cytokines

Serum samples were stored at −80°C until they were ready for analysis. Protein levels were assessed using a Magnetic Luminex Assay (Bio‐Techne, Abingdon, Oxon, UK) and the Magpix Luminex instrument (Luminex Corp, Austin, TX, USA), following the manufacturer's guidelines. The LUMINEX assays included the following cytokines: Adiponectin, matrix metallopeptidase 2 (MMP2), matrix metallopeptidase 9 (MMP9), myeloperoxidase (MPO), myoglobin, carbon tetrachloride (CCL4), chemokine ligand 13 (CXCL13), chemokine ligand 9 (CXCL9), growth/differentiation factor 15 (GDF15), tenascin‐C, galectin, intercellular adhesion molecule 1 (ICAM1), interferon‐gamma (IFN‐γ), interleukin 10 (IL‐10), interleukin 15 (IL‐15), interleukin 17 (IL‐17), interleukin 18 (IL‐18), interleukin 1 receptor antagonist (IL‐1ra), interleukin 6 (IL‐6), interleukin 7 (IL‐7), interleukin 8 (IL‐8), tumour necrosis factor alpha (TNF‐α), and vascular endothelial growth factor (VEGF). The list of their assay ranges is presented in Supporting Material Table S1.

### 2.6 RNA Extraction and qPCR analysis

For the isolation of PBMCs, total RNA was extracted using TRIzol reagent (Invitrogen, Karlsruhe, Germany), following the manufacturer's instructions. RNA concentration and quality were assessed using an Infinite 200 M microplate reader (Tecan, Mainz, Germany) equipped with a NanoQuant plate. The cDNA synthesis was performed as previously described in Keller et al. (2012) and qPCR analysis was conducted on a Rotor‐Gene Q system (Qiagen, Hilden, Germany) with gene‐specific primer pairs (Eurofins MWG Operon, Ebersberg, Germany), as outlined in Chiappisi et al. (2017) and Keller et al. (2012). Primer details are provided in Table 2. To ensure the reliability of the data, qPCR results were normalized using the three most stable reference genes (CANX, MDH1, SDHA), based on the methodology outlined by Vandesompele et al. (2002). Signal transducer and activator of transcription 3 (STAT3), Lamin B1 (LMB1), forkhead box O3 (FOXO3), and tumor protein P53 (TP52) were measured.

**Table 2:** Properties of primers for qPCR analysis.

Gene symbol	NCBI GenBank Accession Number	Primer sequence		Product size (bp)
		forward	reverse	
Reference genes				
*ATP5F1B*	NM_001686	TCGCGTGCCATTGCTGAGCT	CGTGCACGGGACACGGTCAA	218
*SDHA*	NM_004168	CCAAGCCCATCCAGGGGCAAC	TCCAGAGTGACCTTCCCAGTGCCAA	100
*YWHAZ*	NM_003406	TGGGGACTACGACGTCCCTCAA	CATATCGCTCAGCCTGCTCGG	115
Target genes				
*FOXO3*	NM_001455	CTACGAGTGGATGGTGCGTT	TGTGCCGGATGGAGTTCTTC	89
*LMB1*	NM_005573	AGGAGAAGGAGGAGCTGCG	GATTCCTTCTTAGCATAGTTGAGGA	286
*STAT3*	NM_139276	TCTGCCGGAGAAACAGTTGG	AGGTACCGTGTGTCAAGCTG	83
*TP53*	NM_000546	TGTGACTTGCACGTACTCCC	ACCATCGCTATCTGAGCAGC	199

### 2.7 Statistical Analysis

Given the exploratory nature of this study, a formal sample size calculation was not conducted. The sample size of 16 participants was determined based on feasibility and is comparable to previous studies examining mitochondrial function in PBMCs under similar conditions (Zhou et al. 2020). Data were analyzed using SPSS version 26 (IBM, Chicago, IL, USA) and GraphPad Prism (Windows, GraphPad Software, La Jolla, California, USA). Univariate linear regression analysis was performed to determine the associations between mitochondrial respiration (basal and maximal MR_PBMC_) and various independent variables, including PBMC subpopulations, cytokine levels, intracellular signaling markers, and metabolic parameters. For continuous variables, regression coefficients (ß) and their 95% confidence interval (CI) were calculated, and statistical significance was set at *p* < 0.05. The strength of the associations between the variables was determined by evaluating the effect size, with higher ß values indicating stronger relationships. Descriptive statistics are presented as mean ± standard deviation (SD). All tests were two‐tailed, and statistical significance was considered at *p* < 0.05. No adjustments for multiple comparisons were made, reflecting the exploratory nature of the study and its aim to identify broad associations rather than to test specific a priori hypotheses. Multivariate regression models were calculated to assess whether selected associations remained stable after accounting for potential confounders. These models are considered exploratory and serve to complement the univariate results. These models were constructed stepwise: first adjusting for age (Model 1), then sequentially including body fat percentage (Model 2) and VO_2peak_ as covariates to account for lifestyle‐related influences (Model 3). To avoid overfitting, the number of predictors per model was limited, and only variables that were statistically significant or biologically plausible based on univariate results were considered.

## 3 Results

### 3.1 Associations Between MR_PBMC_ and PBMC Subpopulations

**Figure 1:**
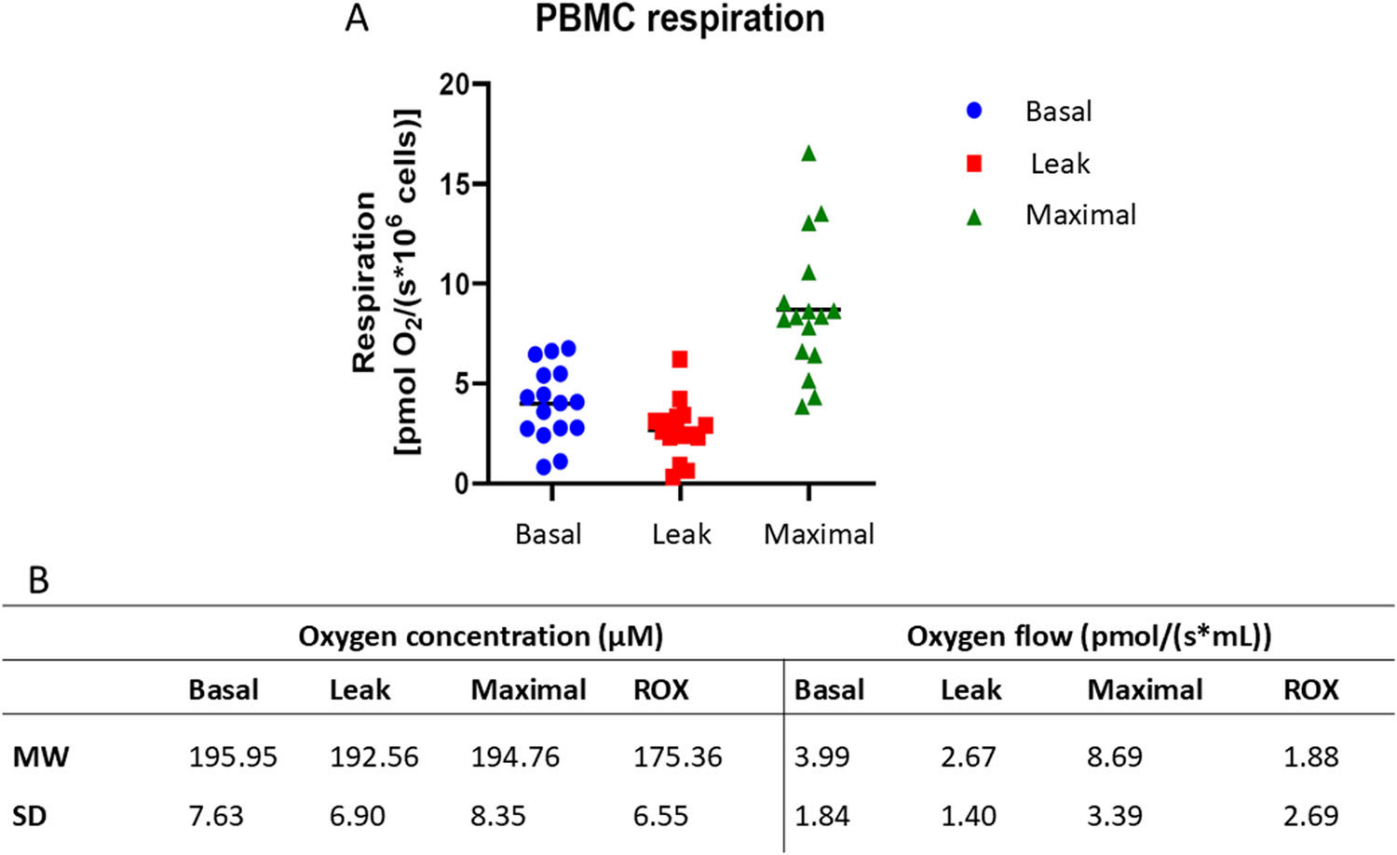
(A) Basal, leak, and maximal respiration in the intact, unstimulated PBMCs. Mitochondrial respiration was measured as basal oxygen consumption in unstimulated PBMCs (“Basal”), after inhibition of complex V (ATPase) with oligomycin (proton leak respiration, “Leak”), and after stepwise titration with the uncoupler FCCP for maximal stimulation of the cells (“Maximal”). (B) Oxygen concentration and flow data in different states. All data are shown in mean ± SD; *n* = 16 participants were used.

The main outcomes for mitochondrial respiration were basal respiration and maximal respiration (see Figure 1). LEAK respiration was not included in the regression analyses but was assessed as a control parameter to evaluate mitochondrial coupling efficiency and membrane integrity. To examine factors that influence MR_PBMC_ univariate linear regression analysis was performed to investigate PBMC subpopulations for their association with MR_PBMC._ Univariate regression revealed associations between basal MR_PBMC_ and proportion of CD4^+^ T cells (ß = 0.093 [0.013–0.173], *p* < 0.05), while maximal MR_PBMC_ was associated with percentage of CD4^+^ (ß = 0.180 [0.033–0.328]), CD8^+^ (0.263 [0.006–0.520]), CD4 naïve (ß = 0.121 [0.026–0.216]), and CD4EM cells (ß = −0.170 [−0.331–−0.009], all *p* < 0.05). No association was found between basal respiration of MR_PBMC_ and proportion of naïve CD4^+^ cells, CD4EM, CD4CM, CD4TEMRA, all CD8^+^ cells, T_eff_, and all T_regs_ (all *p* > 0.05), and between maximal respiration of MR_PBMC_ and proportion of CD4CM, CD4TEMRA, naïve CD8^+^, CD8EM, CD8CM, CD8TEMRA, T_eff_, and all T_regs_ (all *p* > 0.05). More details are shown in Table 3.

**Table 3:** Univariate regression models for mitochondrial respiration of PBMCs and T cell subsets.

Cell type	Basal respiration			Maximal respiration		
	*Adjusted R²*	*ß (95% CI)*	*p* value	*Adjusted R²*	*ß (95% CI)*	*p* value
CD4	0.231	0.093 (0.013–0.173)	**0.025** *	0.251	0.180 (0.033–0.328)	**0.020** *
CD4 naive	−0.017	0.024 (−0.036–0.084)	0.409	0.271	0.121 (0.026–0.216)	**0.016** *
CD4 EM	0.036	−0.057 (−0.150–0.037)	0.218	0.191	−0.170 (−0.331– −0.009)	**0.040** *
CD4 CM	−0.055	−0.014 (−0.104–0.076)	0.746	0.016	−0.087 (−0.0250–0.076)	0.276
CD4TEMRA	−0.037	0.076 (−0.179–0.331)	0.536	0.033	−0.273 (−0.734–0.188)	0.228
CD8	0.062	0.101 (−0.046–0.247)	0.165	0.179	0.263 (0.006–0.520)	**0.046** *
CD8 naive	−0.061	−0.005 (−0.077–0.067)	0.887	0.051	0.083 (−0.045–0.211)	0.187
CD8 EM	0.027	−0.063 (−0.173–0.047)	0.242	0.086	−0.152 (−0.352–0.048)	0.126
CD8 CM	0.124	−0.317 (−0.681–0.047)	0.084	−0.060	−0.063 (−0.814–0.687)	0.860
CD8TEMRA	−0.022	0.029 (−0.048–0.106)	0.438	0.019	0.077 (−0.064–0.219)	0.265
T_eff_	−0.056	0.020 (−0.118–0.157)	0.763	−0.043	−0.065 (−0.321–0.191)	0.596
T_reg_	−0.051	0.122 (−0.483–0.726)	0.675	−0.001	0.519 (−0.586–1.623)	0.334
T_reg_/T_eff_ ratio	−0.050	8.367 (−32.188–48.922)	0.668	0.003	35.921 (−38.081–109.923)	0.319
mT_reg_	−0.060	0.005 (−0.053–0.064)	0.850	−0.051	−0.021 (−0.130–0.088)	0.682
rT_reg_	−0.039	−0.017 (−0.080–0.045)	0.560	−0.057	0.015 (−0.102–0.133)	0.786

Abbreviations: CM, central memory; EM, effector memory; mT_reg_, memory‐T_reg_; T_eff’_, T effector cells; T_reg_, regulatory T cells; rT_reg_, resting‐T_reg_; 95% CI: 95% confidence interval.

* *p* < 0.05.

### 3.2 Association Between MR_PBMC_, Plasma Cytokine Levels, and Intracellular Signaling Molecules

Next, univariate regression analysis was performed to detect associations between MR_PBMC_ and levels of plasma cytokines. Absolute cytokine values are shown in Supporting Material Table S2. Results of regression analysis are shown in Table 4. Basal MR_PBMC_ was negatively associated with both IL‐17 (ß = −0.879 [−1.659 to −0.100]) and TNF‐α (ß = −0.730 [−1.426 to −0.034]) and positively associated with myoglobin (ß = 0.546 [0.016–1.075], all *p* < 0.05). Maximal MR_PBMC_ revealed positive associations to plasma levels of IL‐8 (ß = 0.495 [0.130–0.860] *p* = 0.01), ICAM‐1 (ß = 0.000026 [0.000006–0.000046], *p* = 0.01), and VEGF (ß = 0.027 [0.004–0.049], *p* < 0.05). For intracellular signaling, univariate linear regression was performed, and positive associations between basal respiration and level of STAT3 expression were found (ß = 5.910 [2.073–9.747], *p* < 0.01). No association was found between basal MR_PBMC_ and IL1ra, IL‐6, IL‐7, IL‐8, IL‐10, IL‐15, IL‐18, IFNγ, ICAM‐1, VEGF, Adiponectin, Tenascin‐C, Galectin, MMP2, MMP9, MPO, CCL4, CXCL9, CXCL13, GDF15, STAT3, LMB1, FOXO3, and TP53 (all *p* > 0.05), while no significant association was found between maximal MR_PBMC_ and IL1ra, IL‐6, IL‐7, IL‐10, IL‐15, IL‐17, IL‐18, TNF‐α, IFNγ, Adiponectin, Tenascin‐C, Galectin, MMP2, MMP9, MPO, Myoglobin, CCL4, CXCL9, CXCL13, GDF15, LMB1, FOXO3, and TP53 (all *p* > 0.05) (Table 5).

**Table 4:** Univariate regression models for mitochondrial respiration of PBMCs and cytokines.

Molecule	Basal respiration			Maximal respiration		
	Adjusted R²	ß (95% CI)	*p* value	Adjusted R²	ß (95% CI)	*p* value
IL1ra	0.039	−0.004 (−0.011–0.003)	0.213	−0.023	−0.005 (−0.019–0.009)	0.444
IL‐6	−0.053	−0.069 (−0.403–0.264)	0.663	−0.065	−0.045 (0.−0.671–0.581)	0.880
IL‐7	−0.004	−0.078 (−0.249–0.093)	0.349	−0.020	−0.125 (−0.448–0.199)	0.426
IL‐8	0.016	0.123 (−0.108–0.354)	0.274	0.299	0.495 (0.130–0.860)	**0.011** *
IL‐10	−0.025	−0.686 (−2.589–1.218)	0.456	−0.059	−0.418 (−4.042–3.205)	0.810
IL‐15	0.094	−0.173 (−0.393–0.048)	0.116	0.055	−0.280 (−0.702–0.141)	0.177
IL‐17	0.217	−0.879 (−1.659 to −0.100)	**0.029** *	0.138	−1.396 (−2.928–0.136)	0.071
IL‐18	−0.060	0.00005 (−0.00044–0.00053.)	0.843	−0.061	−0.00006 (−0.00096–0.00085)	0.893
TNF‐α	0.118	−0.730 (−1.426–−0.034)	**0.041** *	0.017	−0.769 (−2.204–0.666)	0.273
IFNγ	0.153	−0.011 (−0.025–0.002)	0.083	0.136	−0.021 (−0.046–0.004)	0.097
ICAM‐1	−0.014	0.000005 (−0.000007–0.000018)	0.397	0.282	0.000026 (0.000006–0.000046)	**0.014** *
VEGF	0.003	0.007 (−0.007–0.020)	0.321	0.244	0.027 (0.004–0.049)	**0.022** *
Adiponectin	−0.060	0.046 (−0.492–0.584)	0.858	−0.049	0.216 (−0.786–1.217)	0.654
Tenascin‐C	−0.036	−0.364 (−1.579–0.850)	0.534	0.014	1.164 (−0.1055–3.383)	0.283
Galectin	−0.057	0.169 (−1.075–1.412)	0.777	−0.059	−0.234 (−2.566–2.097)	0.834
MMP2	−0.057	0.008 (−0.049–0.064)	0.779	0.004	0.050 (−0.053–0.153)	0.317
MMP9	0.061	−0.008 (−0.019–0.004)	0.116	0.033	−0.012 (−0.034–0.009)	0.228
MPO	0.148	−0.027 (−0.055–0.002)	0.064	−0.020	−0.022 (−0.081–0.036)	0.429
Myoglobin	0.182	0.546 (0.016–1.075)	**0.044**	0.054	0.706 (−0.360–1.771)	0.180
CCL4	0.086	−0.021 (−0.048–0.007)	0.127	0.033	−0.031 (−0.084–0.022)	0.227
CXCL9	0.015	−0.002 (−0.006–0.002)	0.279	0.075	−0.005 (−0.013–0.002)	0.142
CXCL13	−0.010	−0.021 (−0.069–0.028)	0.374	−0.062	−0.004 (−0.097–0.089)	0.933
GDF15	−0.013	0.00009 (−0.00013 to 0.00031)	0.389	0.017	0.00022 (−0.00019–0.00063)	0.271

Abbreviations: IL, interleukin; CCL4, carbon tetrachloride; CXCL, chemokine ligand; GDF15, growth/differentiation factor 15; ICAM1, intercellular adhesion molecule 1; MMP, matrix metallopeptidase; MPO, myeloperoxidase; TNF‐α, tumour necrosis factor alpha; IFNγ, interferon‐gamma; VEGF, vascular endothelial growth factor; 95% CI, 95% confidence interval.

* *p* < 0.05.

**Table 5:** Univariate regression models for mitochondrial respiration of PBMCs and intracellular signaling markers.

Gene	Basal respiration			Maximal respiration		
	*Adjusted R²*	*ß (95% CI)*	*p* value	*Adjusted R²*	*ß (95% CI)*	*p* value
STAT3	−0.43	0.831 (−1.746–3.408)	0.493	0.466	5.910 (2.073–9.747)	**0.006** *
LMB1	0.113	1.750 (−0.672–4.173)	0.140	0.042	2.945 (−2.296–8.185)	0.242
FOXO3	0.003	−0.724 (−2.293–0.845)	0.331	−0.085	0.382 (−3.024–3.789)	0.809
TP53	−0.099	−0.063 (−1.431–1.305)	0.920	−0.028	1.106 (−1.838–4.051)	0.422

Abbreviations: FOXO3, forkhead box O3; LMB1, Lamin B1; STAT3, signal transducer and activator of transcription 3; TP53, tumor protein P53; 95% CI, 95% confidence interval.

* *p* < 0.05.

### 3.3 Association of MRr_PBMC_ to Anthropometric Data, Cardiorespiratory Fitness, and Metabolic Risk Factors

Likewise, univariate linear regression was performed with MR_PBMC_ and anthropometric, fitness, and metabolic risk factors (see Table 6). Here, basal MR_PBMC_ was positively associated with age (ß = 0.260 [0.048–0.473], *p* < 0.05), levels of blood glucose (ß = 0.104 [0.0021–0.186], *p* < 0.05), while maximal MR_PBMC_ was associated with glucose (ß = 0.194 [0.039–0.348], *p* < 0.05) and HbA1c (ß = −5.777 [−11.277 to −0.277], *p* < 0.05). No association was found between basal MR_PBMC_ and BMI, body fat, VO_2peak_, hand grip strength, and HbA1c (all *p* > 0.05), while no significant association was found between maximal MR_PBMC_ and age, BMI, body fat, VO_2peak_, and hand grip strength (all *p* > 0.05).

**Table 6:** Univariate regression models for mitochondrial respiration of PBMCs and anthropometric data.

Anthropometrics	Basal respiration			Maximal respiration		
	*Adjusted R²*	*ß (95% CI)*	*p* value	*Adjusted R²*	*ß (95% CI)*	*p* value
Age	0.253	0.260 (0.048–0.473)	**0.019** *	−0.022	0.175 (−0.290–0.641)	0.437
BMI	−0.047	0.074 (−0.254–0.402)	0.639	−0.044	0.156 (−0.457–0.769)	0.597
Body fat (%)	−0.030	−0.47 (−0.188–0.094)	0.491	0.001	0.123 (0.123)	0.329
VO_2peak_	−0.029	0.034 (−0.067–0.136)	0.483	−0.060	−0.019 (−0.212–0.175)	0.841
Hand grip strength	0.030	0.023 (−0.017–0.063)	0.236	−0.055	0.012 (−0.090–0.066)	0.748
Glucose	0.264	0.104 (0.021–0.186)	**0.017** *	0.263	0.194 (0.039–0.348)	**0.017** *
HbA1c	−0.046	−0.784 (−4.119–2.551)	0.625	0.189	−5.777 (−11.277 to −0.277)	**0.041** *

Abbreviation: 95% CI: 95% confidence interval.

* *p* < 0.05

### 3.4 Exploratory Multivariate Regression Analyses

To further examine the robustness of the observed associations, we performed exploratory multivariate regression analyses adjusting stepwise for covariates. Detailed regression results for all models, including unstandardized coefficients, 95% CI, p‐values, and adjusted R² values, are provided in Supporting Material Table S3. We first controlled for age alone, and subsequently added body fat % and VO₂_peak_ to assess the potential influence of lifestyle‐related factors. For basal MR_PBMC_, the strongest model included CD4⁺ T cell percentage and age (adjusted R² = 0.436, *p* = 0.005), with both variables showing significant associations (CD4⁺: ß = 0.081, 95% CI [0.012, 0.150], *p* = 0.025; age: ß = 0.229, 95% CI [0.042, 0.417], *p* = 0.020). Similarly, TNF‐α (ß = –0.703, 95% CI [–1.279, –0.127], *p* = 0.020) and age (ß = 0.253, 95% CI [0.069, 0.436], *p* = 0.010) were both independently associated in their model (adjusted R² = 0.450, *p* = 0.004). Age (ß = 0.207, 95% CI [0.000, 0.413], *p* = 0.050) also emerged as the sole significant predictor in the IL‐17 model (adjusted R² = 0.360, *p* = 0.014), whereas IL‐17 itself was not independently associated. In addition, fasting glucose remained significantly associated with basal MR_PBMC_ after full adjustment (ß = 0.092, 95% CI [0.007, 0.177], *p* = 0.036; adjusted R² = 0.373, *p* = 0.037). In contrast, for maximal mitochondrial respiration, CD4⁺ T and naïve CD4⁺ T cells showed the strongest univariate associations, which were not improved by adding lifestyle‐related covariates. In contrast, CD4⁺ effector memory T cells remained significantly associated in a multivariate model including age and body fat (ß = –0.200, 95% CI [–0.353, –0.046], *p* = 0.015; adjusted R² = 0.309, *p* = 0.043). IL‐8 was significantly associated with basal MR_PBMC_ (ß = 0.481, 95% CI [0.095, 0.867], *p* = 0.018), independently of age. Likewise, ICAM‐1 and VEGF were the only independent predictors in their univariate models. CD8⁺ T cells also showed independent explanatory value in the univariate model only. HbA1c was inversely associated with maximal MR_PBMC_ and remained the only significant predictor in the adjusted model 2 (ß = –6.865, 95% CI [–12.141, –1.589], *p* = 0.014; adjusted R² = 0.310, *p* = 0.043). Fasting glucose also showed a positive univariate association with maximal MR_PBMC_, but this did not remain robust in multivariate models. Notably, the model including STAT3, age, body fat, and VO₂_peak_ yielded the best overall fit for maximal MR_PBMC_ (adjusted R² = 0.495, *p* = 0.047), with STAT3 as the sole significant predictor (ß = 4.992, 95% CI [0.450, 9.534], *p* = 0.035). No significant contribution was observed for VO₂_peak_ or body composition in any of the models.

## 4 Discussion

Our results highlight how mitochondrial respiration of PBMCs is related to the proportion of CD4^+^ T cell subsets, systemic inflammation, and activation of intracellular signaling pathways. In particular, our findings demonstrate that increased mitochondrial respiration is associated with both a more favorable distribution of CD4⁺ T cell subtypes, especially naive T cells, and lower concentrations of pro‐inflammatory cytokines such as TNF‐α and IL‐17. Furthermore, the observed positive correlations between maximal mitochondrial respiration and vascular markers such as ICAM‐1 and VEGF demonstrate how mitochondrial bioenergetics might affect both immune and vascular function. These findings emphasize the central role of mitochondrial function as a potential key factor in promoting healthy aging.

Age‐associated changes in the functionality of immune cells, particularly in subtypes of PBMCs, and their link to chronic inflammation and decreased immunity has already been researched from different perspectives, but the metabolic consequences for mitochondrial respiration remained unclear (Miwa et al. 2022). In this healthy elderly cohort, the whole CD4^+^ T cell pool was positively associated with basal and maximal MR_PBMC_. Herein, a higher percentage of naïve CD4^+^ T cells was associated with an increased maximal MR_PBMC_. These associations remained robust after adjusting for age, body fat, and VO₂_peak_. These findings are consistent with data from other studies, which demonstrate that CD4^+^ T cells from older adults exhibit elevated basal and maximal oxygen consumption rates (Bharath et al. 2020). Previous data by Withnall et al. (2024) demonstrated a higher mitochondrial dependence of CD4^+^ T cells, naïve CD8^+^, EM, and CM T cells in older adults, whereas CD4^+^ T cells in younger adults showed a greater reliance on glycolytic metabolism. The proportion of naïve CD4^+^ T cells tends to decrease within aging, while effector and memory cells increase (Boßlau et al. 2021). Therefore, only a change in the composition of the PBMC subpopulations due to differences in metabolic performance can influence overall respiratory performance (Hodgman et al. 2023). Nevertheless, there is also evidence of impaired mitochondrial respiration, including basal and maximal respiration in CD4^+^ T cells in older adults (Bektas et al. 2019). In this regard, an accumulation of mitochondrial proteins in CD4^+^ T cells from older adults was found compared to younger adults suggesting a lack of mitochondrial recycling due to defective autophagy (Bektas et al. 2019; Sriwichaiin et al. 2023). In addition, CD8^+^ T cells also undergo age‐related changes in both their composition and function. As individuals age, the number of naïve CD8^+^ T cells decreases, while the proportion of memory and effector cells increases (Ponnappan and Ponnappan 2011). These shifts not only impact the immune response but also influence the metabolic profile of CD8^+^ T cells.

In our study, the percentage of CD4^+^EM cells was associated with lower maximal MR_PBMC_. Even after accounting for age and body fat in multivariate models, CD4⁺EM T cells remained independently associated with reduced maximal respiration. This is contrasted by findings that in adults of different age, memory CD4^+^ T cells had higher oxidative phosphorylation than naïve CD4^+^ T cells due to the increase of CPT1a expression. Following a polyclonal stimulation, CD4^+^ memory T cells from older adults demonstrated increased mitochondrial activity (OXPHOS), including higher ROS and ATP levels, independent of activation‐induced mitochondrial biogenesis. While those aged CD4^+^ memory T cells maintained a more catabolic lipid metabolism, these cells retained the ability to enhance glycolysis upon activation (Yanes et al. 2019).

Aging displays a state of chronic low‐grade inflammation with increased serum levels of inflammatory markers. Research in elderly people has shown a decreased frequency of IL‐17‐producing cells in the memory subset of CD4^+^ T cells compared to younger people (Coperchini et al. 2024). In general, levels of IL‐17 released by various immune cells such as NK cells or neutrophils increase with age and in particular CD4^+^ T cells from healthy older people preferentially develop a Th17 profile (Bharath et al. 2020; Xu et al. 2024). In our healthy elderly cohort, IL‐17 was inversely associated with basal mitochondrial respiration in univariate analyses; however, this relationship did not persist after adjusting for age. This suggests that the observed association likely reflects age‐related immunological shifts rather than a direct metabolic link. Consequently, IL‐17 may serve more as a marker of immune system remodeling than as a determinant of mitochondrial function in this population (Huangfu et al. 2023). Potential mechanisms, such as suppressed glycolysis—relevant for IL‐17 production—or enhanced mitochondrial resilience through reduced inflammatory signaling, remain speculative in this context.

The observed relationship between inflammation and mitochondrial respiration further highlights the role of TNF‐α, a key cytokine for overall inflammatory responses, whose signaling pathway activation represents a biomarker of cellular senescence (Bao et al. 2023). Within our healthy aging cohort, serum TNF‐α was negatively associated with the basal MR_PBMC_. This association persisted after adjustment for age, supporting the role of TNF‐α as an independent marker of reduced mitochondrial function. TNF‐α drives inflammation and ensures immune cells’ activation following a change in cellular energy metabolism (Bao et al. 2023). However, the data from the literature are very heterogeneous. Various studies show that TNF‐α is chronically elevated to a slight degree in old age (Brüünsgaard and Pedersen 2003; P. Li et al. 2015). In early‐stage heart failure patients, a negative correlation between the mitochondrial respiratory function and TNF‐α, IL‐6, CRP, and cardiometabolic risk factors was found, while the basal and maximal MR_PBMC_ was reduced compared to a healthy control group (P. Li et al. 2015). Although the role of TNF‐α in the aging process continues to be widely discussed, our results suggest that improved mitochondrial function–recognizable by higher basal MR_PBMC_–could attenuate inflammatory processes. This is supported by the lower TNF‐α serum levels observed in our cohort. However, we can only show a connection, not causality. Accordingly, it is also conceivable that TNF‐α conversely inhibits oxidative metabolism.

Cellular senescence is a central mechanism for the development of cardiovascular and atherosclerotic diseases due to disturbed vascular endothelial function (Hu et al. 2022). ICAM‐1 is expressed by the vascular endothelium, monocytes/macrophages, and lymphocytes and is demonstrated to be increased in both, healthy older adults and patients with pathological diseases, including hypertension, diabetes, and atherosclerosis (Dougherty et al. 1988; Van der Meer et al. 2002). In our healthy, older study cohort, we found a positive association between maximal MR_PBMC_ and ICAM‐1 serum levels independent of lifestyle covariates. One the one hand, an increased maximal MR_PBMC_ may serve as a mechanism to counteract oxidative stress or age‐related inflammatory processes, on the other hand, a higher mitochondrial capacity might reflect an improved ability to support immune responses through i.e. ICAM‐1‐mediated cellular dynamics.

In addition, intracellular signaling showed associations with mitochondrial respiration, in our study with STAT signaling. Within aging, a relation to progressively higher levels of STAT3 signaling activity was previously found in PBMCs of older adults. Here, higher STAT3 was suggested to be driven by age‐related increases of systemic inflammation (Piber et al. 2019). Furthermore, there is evidence that aging promotes localization of STAT3 in the mitochondria of CD4^+^ T cells, leading to changes in the structure and function of mitochondria, which affects mitochondrial dynamics and cytokine production (Zukowski et al. 2023). The positive correlation between maximal MR_PBMC_ and STAT3 suggests that STAT3 may enhance mitochondrial capacity either directly within mitochondria or indirectly via transcriptional regulation. Among all variables tested, STAT3 exhibited the strongest and most consistent association with maximal mitochondrial respiration, independent of all covariates. This highlights its key role in linking cellular energy metabolism and immune function. Similarly, VEGF and IL‐8 exhibit notable connections to mitochondrial respiration and inflammation. VEGF, known for its key function as a growth factor in angiogenesis, vascular maintenance, and inflammation, has been shown to enhance mitochondrial function in endothelial cells and mitochondria play a dominant role in the stimulation of VEGF (D. Guo et al. 2017).

Our findings reveal a positive association between maximal MR_PMBC_, serum VEGF, and serum IL‐8 levels. In multivariate analyses, both VEGF and IL‐8 remained independently associated with maximal respiration. IL‐8, a potent, proangiogenic, and inflammatory chemokine, has been shown to upregulate VEGF expression in endothelial cells (Martin et al. 2009). The mechanistic role of STAT3 in enhancing mitochondrial capacity may underpin this relationship, as greater mitochondrial efficiency could meet the energy demands of processes mediated by VEGF and IL‐8. VEGF's association with MR_PBMC_ may indicate its involvement not only in oxygen delivery but also in supporting cellular energy needs, potentially optimizing PBMC function under mitochondrial stress. Similarly, enhanced mitochondrial capacity could improve PBMC responsiveness to IL‐8 signaling, particularly during inflammatory challenges. These associations might reflect improved vascularization, immune competence, and enhanced overall fitness in a healthy aging cohort. However, the absence of correlations between MR_PBMC_ and fitness parameters, despite VO_2peak_ values aligning with ACSM standards, may stem from the cohort's homogeneity and lack of disease burden (Riebe et al. 2018). This is further supported by the lack of independent associations between VO₂_peak_ and mitochondrial respiration in any of the multivariate models.

A healthy and active lifestyle has been a key research focus in recent decades, particularly regarding its impact on mitochondrial respiration in immune cells. Cardiorespiratory fitness (CRF), typically assessed as VO_2peak_, has been shown to influence mitochondrial respiration in PBMCs, altering their transcriptomic and metabolic profile (Gebhardt et al. 2024). Various studies showed that interventions including physical activity and exercise over weeks are followed by mitochondrial capacity changes towards a higher maximal respiration (Krüger and Gebhardt 2024). Studies on active older adults and lifelong exercisers suggest a reduced inflammatory state compared to their sedentary counterparts, potentially mediated by enhanced mitochondrial function (Pérez‐Castillo et al. 2025). In our healthy aging cohort, basal MR_PBMC_ showed a positive association with age, supporting the hypothesis of increased mitochondrial dependence in aging. However, the relationship between mitochondrial respiration and age remains inconclusive due to heterogeneous findings in the literature (Ehinger et al. 2024).

Aging is also associated with metabolic changes that can affect glucose homeostasis. Impairments in insulin sensitivity and glucose regulation are well‐documented in aging populations, often linked to alterations in mitochondrial function. A possible connection between these processes is reflected in our findings. The negative association between MR_PBMC_ and HbA1c levels could indicate metabolic dysfunction in the context of impaired glucose homeostasis. There is also evidence for this in PBMCs, which has already been linked to altered gene expression in those, particularly in genes involved in cell cycle processes (Slieker et al. 2018).

These findings point to mitochondrial respiration in PBMCs as a potential target for future interventions aimed at supporting immune resilience in aging. Approaches such as physical activity may modulate mitochondrial bioenergetics and thereby contribute to a more balanced immune phenotype, but this requires validation in longitudinal or interventional studies. However, given the exploratory character of this study, several limitations must be considered when interpreting the results. First, the small sample size limits statistical power, increasing the risk of Type II errors, particularly for small to moderate associations. Second, multiple univariate analyses were performed without adjustments for multiple comparisons, reflecting the exploratory nature of the study aimed at identifying broad patterns rather than testing predefined hypotheses. Third, although exploratory multivariate models were used to adjust for potential confounders of main findings, their results should be interpreted cautiously. Consequently, findings should be seen as hypothesis‐generating and require validation in larger cohorts. Fourth, subgroup sizes were too small for stratified analyses by sex. Future studies with sufficient power are needed to address potential sex‐specific differences. Fifth, the healthy cohort, consisting of participants free of diagnosed diseases and medications, differs from many prior studies that included individuals with pre‐existing conditions or treatments that could influence immune or mitochondrial function. Finally, the cross‐sectional design limits causal inferences, as observed associations reflect correlations at a single time point. Additionally, while some studies report that cryopreservation time does not adversely affect mitochondrial function, others suggest that samples should ideally be analyzed within a week post‐freezing due to declining respiratory capacities (Dieter et al. 2023; Werner et al. 2022). More recently, Payne et al. (2025) demonstrated that mitochondrial function can be preserved for up to 3 months at –80°C, but since our PBMCs were stored for up to 6 months, potential alterations due to prolonged cryopreservation cannot be excluded. It is important to note that we did not assessed mitochondrial mass, so our findings represent cell‐based respiratory capacity rather than mitochondria‐specific function. This distinction is critical, as differences in respiration profiles between groups may reflect variations in mitochondrial content rather than solely cell‐based capacity, and such respiration rates may mask underlying deficits unless normalized to mtDNA copy number (Alonso et al. 2021; Sartori et al. 2025). Future studies with larger and more diverse cohorts, applying longitudinal designs and adequately powered multivariate analyses, are needed to validate and extend these findings. They should include normalization per mitochondrion to enable more precise interpretation of mitochondrial function.

## 5 Conclusion

In conclusion, this study highlights significant associations between mitochondrial respiration in PBMCs and key immunological, inflammatory, and metabolic markers in a healthy elderly cohort. Notably, higher mitochondrial respiration was positively associated with a more favorable distribution of CD4⁺ T cell subsets, particularly naïve T cells, which are indicative of robust immune function. Conversely, elevated levels of pro‐inflammatory cytokines, such as TNF‐α were inversely associated with basal mitochondrial respiration, suggesting a potential link between chronic low‐grade inflammation and impaired mitochondrial function during aging. These findings emphasize the importance of mitochondrial function as a critical determinant of immunometabolic health in aging populations. Future studies should further investigate the mechanistic pathways linking mitochondrial bioenergetics, immune cell metabolism, and healthy aging, while exploring interventions ‐ such as physical activity and targeted lifestyle modifications ‐ that could preserve mitochondrial function and promote successful aging.

## Author Contributions

**Kristina Gebhardt:** conceptualization, development and design of methodology, investigation, formal analysis, writing‐ original draft, visualization, writing – review and editing. **Anne Hebecker:** investigation. **Magdalena Huber:** conceptualization, methodology, funding acquisition. **Hartmann Raifer:** methodology, validation, investigation. **Natascha Sommer:** conceptualization, methodology, funding acquisition. **Robert Ringseis:** conceptualization, methodology, validation, investigation, writing – review and editing, funding acquisition. **Klaus Eder:** conceptualization, writing – review and editing, funding acquisition. **Karsten Krüger:** conceptualization, methodology, writing – original draft, writing – review and editing, supervision, funding acquisition. **Christopher Weyh:** conceptualization, methodology, validation, investigation, formal analysis, writing – original draft, writing – review and editing.

## Ethics Statement

The study was approved by the Medical Ethics Committee of Justus‐Liebig University Giessen (AZ 100/20). All procedures were conducted in line with the Declaration of Helsinki.

## Consent

Written informed consent was obtained from all participants before their inclusion in the study.

## Conflicts of Interest

The authors declare no conflicts of interest.

## Supporting information


**Figure S1:** Typical trace from intact peripheral blood mononuclear cells (PBMCs) from older participants using Oxygraph‐2k.

**Table S1:** Cytokine ranges and Detection rates. Table S2. Absolute values of serum cytokines. Table S3: Multivariate regression model.

## Data Availability

All data are available upon request. The data that support the findings of this study are available on request from the corresponding author upon reasonable request.
