# Supplementary material for: PBMCs Mitochondrial Respiration and Its Relation to Immunity, Fitness, and Metabolic Risk in the Healthy Elderly
Source: J Cell Physiol. 2025 Sep 27;240(9):e70096. doi: 10.1002/jcp.70096 (PMC12475958; doi:10.1002/jcp.70096)
Supplement: Supplementary file 1 — Figure S1: Typical trace from intact peripheral blood mononuclear cells (PBMCs) from older participants using Oxygraph‐2k. [file JCP-240-0-s001.docx]

**Figure S1. Typical trace from intact peripheral blood mononuclear cells (PBMCs) from older participants using Oxygraph-2k.** The SUIT protocol consisted of sequential titration of multiple substrates (left y-axis, oxygen concentration in the chamber; right y-axis, oxygen flux; x-axis, time in h and min; ce2Omy, ATP synthase inhibitor Oligomycin, ce3U*, uncoupling agent FCCP, ce4Ama, complex III inhibitor antimycin A). The red line represents the cell-specific O2 flow, calculated as the negative time derivative of the O2 concentration, expressed as pmol/s/10^6 cells.
