# Supplementary material for: PBMCs Mitochondrial Respiration and Its Relation to Immunity, Fitness, and Metabolic Risk in the Healthy Elderly
Source: J Cell Physiol. 2025 Sep 27;240(9):e70096. doi: 10.1002/jcp.70096 (PMC12475958; doi:10.1002/jcp.70096)
Supplement: Supplementary file 2 — Table S1: Cytokine ranges and Detection rates. Table S2. Absolute values of serum cytokines. Table S3: Multivariate regression model. [file JCP-240-0-s002.docx]

**Table S1.** **Cytokine ranges and Detection rates.**

| Marker | Standard Curve (pg/mL) | Detection rate |
| --- | --- | --- |
| Adiponectin/Acrp30 | 823-200000 | 100% |
| CCL4/MIP-1beta | 123 - 30000 | 100% |
| CXCL9/MIG | 617 - 150000 | 100% |
| CXCL13/BLC/BCA-1 | 18.5 - 4500 | 100% |
| Galectin-3 | 16.9 - 4100 | 100% |
| GDF-15 | 18.5 - 4500 | 100% |
| ICAM-1/CD54 | 7000 - 1700000 | 100% |
| IFN-gamma | 2.67 - 650 | 86% |
| IL-1ra/IL-1F3 | 28.8 - 7000 | 98% |
| IL-6 | 3.25 - 790 | 98% |
| IL-7 | 5.14 - 1250 | 100% |
| IL8/CXCL8 | 4.12 - 1000 | 100% |
| IL-10 | 1.89 - 460 | 100% |
| IL-15 | 6.3 - 1550 | 100% |
| IL-17/IL17A | 13.2 - 3200 | 100% |
| IL-18/IL-1F4 | 13.9 - 3380 | 100% |
| MMP-2 | 276 - 67000 | 100% |
| MMP-9 | 123 - 30000 | 100% |
| Myeloperoxidase/MPO | 123 - 30000 | 100% |
| Myoglobin | 15.4 - 3800 | 100% |
| Tenascin C | 53.5 - 13000 | 100% |
| TNF-alpha | 8.23 - 2000 | 100% |
| VEGF | 11.5 - 2800 | 100% |

**Table S2. Absolute values of serum cytokines.**

| Cytokine | MW (SD) | Minimum | Maximum |
| --- | --- | --- | --- |
| IL1-ra | 408.40 (120.82) | 230.77 | 711.54 |
| IL-6 | 1.77 (3.10) | 0.11 | 12.19 |
| IL-7 | 11.25 (5.36) | 5.73 | 23.37 |
| IL-8 | 6.15 (3.88) | 1.29 | 14.16 |
| IL-10 | 0.86 (0.50) | 0.31 | 2.27 |
| IL-15 | 4.60 (4.08) | 1.78 | 16.38 |
| IL-17 | 1.21 (1.06) | 0.59 | 5.08 |
| IL-18 | 4735.76 (1938.06) | 2034.34 | 8894.73 |
| IFN-gamma | 45.60 (77.55) | 0.84 | 286.55 |
| TNF-alpha | 3.96 (1.21) | 2.48 | 6.56 |
| ICAM1 | 99,404 (74,176.96) | 11562.22 | 279,676.03 |
| VEGF | 85.02 (66.67) | 7.66 | 271.88 |
| Adiponectin | 7.67 (1.76) | 5.45 | 11.40 |
| Tenascin-C | 2.49 (0.79) | 1.08 | 3.75 |
| Galectin | 3.25 (0.71) | 2.02 | 4.23 |
| MMP2 | 195.60 (16.76) | 152.56 | 218.68 |
| MMP9 | 168.96 (81.94) | 55.75 | 316.67 |
| MPO | 72.96 (30.49) | 23.22 | 114.02 |
| Myoglobin | 4.44 (1.59) | 2.70 | 8.69 |
| CCL4 | 123.80 (32.39) | 83.64 | 191.19 |
| CXCL9 | 452.96 (225.48) | 300.32 | 1030.46 |
| CXCL13 | 31.24 (18.93) | 3.75 | 68.52 |
| GDF15 | 13,126.21 (4315.77) | 5905.45 | 24,511.87 |

**Table S3: Multivariate regression model**

|  | | | Model 1 | | | Model 2 | | | Model 3 | | |
| --- | --- | --- | --- | --- | --- | --- | --- | --- | --- | --- | --- |
|  | *Adj. R² (p-value)* | *ß (95% CI)* | | *p-value* | *Adj. R² (p-value)* | *ß (95% CI)* | *p-value* | *Adj. R²*  *(p-value)* | | *ß (95% CI)* | *p-value* |
| CD4 - basal | **0.436 (0.005)** | **0.081 (0.012 – 0.150)** | | **0.025** | **0.416 (0.014)** | **0.083 (0.012 – 0.154)** | **0.026** | **0.378 (0.035)** | | **0.082 (0.007 – 0.156)** | **0.034** |
| Age |  | **0.229 (0.042-0.417)** | | **0.020** |  | **0.219 (0.024 – 0.414)** | **0.030** |  | | **0.233 (0.016 – 0.449)** | **0.037** |
| Body fat (%) |  |  | |  |  | -0.036 (-0.145 – 0.74) | 0.497 |  | | -0.062 (-0.248 – 0.123) | 0.480 |
| VO_2peak_ |  |  | |  |  |  |  |  | | -0.026 (-0.167 – 0.115) | 0.698 |
| CD4 - maximal | 0.218 (0.062) | **0.174 (0.021 – 0.327)** | | **0.028** | 0.228 (0.087) | 0.168 (0.014 – 0.321) | 0.035 | 0.228 (0.119) | | 0.175 (0.019 – 0.330) | 0.030 |
| Age |  | 0.109 (-0.305 – 0.522) | | 0.584 |  | 0.144 (-0.275 – 0.564) | 0.472 |  | | 0.071 (-0.381 – 0.523) | 0.740 |
| Body fat (%) |  |  | |  |  | 0.120 (-0.115 – 0.355) | 0.291 |  | | 0.261 (-0.126 – 0.648) | 0.169 |
| VO_2peak_ |  |  | |  |  |  |  |  | | 0.135 (-0.159 – 0.249) | 0.338 |
| CD4 naive - maximal | **0.253 (0.044)** | **0.119 (0.022 – 0.216)** | | **0.019** | 0.276 (0.058) | 0.116 (0.020 – 0.212) | **0.021** | 0.221 (0.125) | | 0.115 (0.011 – 0.218) | 0.032 |
| Age |  | 0.147 (-0.254 – 0.548) | | 0.445 |  | 0.184 (-0.219 – 0.586) | 0.344 |  | | 0.174 (-0.273 – 0.620) | 0.415 |
| Body fat (%) |  |  | |  |  | 0.129 (-0.098 – 0.356) | 0.244 |  | | 0.150 (-0.249 – 0.548) | 0.432 |
| VO_2peak_ |  |  | |  |  |  |  |  | | 0.020 (- 0.283 – 0.322) | 0.891 |
| CD4 EM - maximal | 0.185 (0.084) | -0.171 (-0.334 – -0.009) | | 0.040 | **0.309 (0.043)** | **-0.200(-0.353 – -0.046)** | **0.015** | 0.277 (0.083) | | -0.197 (-0.356 – -0.038) | 0.019 |
| Age |  | 0.184 (-0.235 – 0.602) | | 0.364 |  | 0.241 (-0.152 – 0.634) | 0.209 |  | | 0.198 (0.-0.233 – 0.629) | 0.339 |
| Body fat (%) |  |  | |  |  | 0.203 (-0.024 – 0.429) | 0.075 |  | | 0.288 (-0.087 – 0.664) | 0.121 |
| VO_2peak_ |  |  | |  |  |  |  |  | | 0.082 (-0.201 – 0.365) | 0.542 |
| CD8 - maximal | 0.172 (0.095) | 0.265 (0.006 – 0.525) | | 0.046 | 0.149 (0.162) | 0.243 (-0.029 – 0.515) | 0.076 | 0.122 (0.235) | | 0.246 (-0.033 – 0.524) | 0.079 |
| Age |  | 0.184 (-0.237 – 0.605) | | 0.366 |  | 0.208 (-0.227 – 0.644) | 0.322 |  | | 0.151 (-0.323 – 0.625) | 0.503 |
| Body fat (%) |  |  | |  |  | 0.090 (-0.162 – 0.343) | 0.455 |  | | 0.205 (-0.210 – 0.620) | 0.306 |
| VO_2peak_ |  |  | |  |  |  |  |  | | 0.110 (-0.202 – 0.422) | 0.460 |

|  | Model 1 | | | Model 2 | | | Model 3 | |  |
| --- | --- | --- | --- | --- | --- | --- | --- | --- | --- |
|  | *Adj. R² (p-value)* | *ß (95% CI)* | *p-value* | *Adj. R² (p-value)* | *ß (95% CI)* | *p-value* | *Adj. R²*  *(p-value)* | *ß (95% CI)* | *p-value* |
| IL-8 - maximal | **0.259 (0.041)** | **0.481 (0.095 – 0.867)** | **0.018** | 0.207 (0.104) | 0.467 (0.017 – 0.917) | 0.043 | 0.308 (0.065) | 0.596 (0.143 – 1.048) | 0.014 |
| Age |  | 0.069 (-0.338 – 0.477) | 0.721 |  | 0.078 (-0.363 – 0.518) | 0.711 |  | -0.084 (-0.545 – 0.376) | 0.698 |
| Body fat (%) |  |  |  |  | 0.018 (-0.248 – 0.284) | 0.885 |  | 0.234 (-0.132 – 0.600) | 0.191 |
| VO_2peak_ |  |  |  |  |  |  |  | 0.238 (-0.057 – 0.534) | 0.105 |
| IL-17 - basal | **0.360 (0.014)** | -0,666 (-1.406 – 0.074) | 0.074 | 0.316 (0.040) | -0.689 (-1.506 – 0.128) | 0.092 | 0.263 (0.092) | -0.690 (-1.598 – 0.218) | 0.125 |
| Age |  | **0.207 (0.001 – 0.413)** | **0.05** |  | 0.208 (-0.007 – 0.423) | 0.057 |  | 0.208 (-0.041 – 0.456) | 0.094 |
| Body fat (%) |  |  |  |  | 0.011 (-0.114 – 0.135) | 0.858 |  | 0.011 (-0.216 – 0.238) | 0.918 |
| VO_2peak_ |  |  |  |  |  |  |  | 0.001 (-0.162 - 0.163) | 0.995 |
| TNF-α - basal | **0.450 (0.004)** | **-0.703 (-1.279 –**  **-0.127)** | **0.020** | **0.418 (0.014)** | **-0.743 (-1.378 –**  **-0.109)** | **0.025** | **0.416 (0.024)** | **-0.780 (-1.425 –**  **-0.135)** | **0.022** |
| Age |  | **0.253 (0.069 – 0.436)** | **0.010** |  | **0.258 (0.066 – 0.451)** | **0.012** |  | **0.291 (0.084 – 0.498)** | **0.009** |
| Body fat (%) |  |  |  |  | 0.021 (-0.094 – 0.137) | 0.696 |  | -0.042 (-0.222 – 0.139) | 0.628 |
| VO_2peak_ |  |  |  |  |  |  |  | -0.062 (-0.199 – 0.075) | 0.343 |
| ICAM-1 - maximal | **0.247 (0.047)** | **0.000025 (0.000004 –0.000046 )** | **0.020** | 0.285 (0.054) | 0,000025 (0.000005 – 0.000045 ) | 0.019 | 0.238 (0.111) | 0,000027  (0.000003 - 0,000050) | 0.028 |
| Age |  | 0.098 (-0.308 – 0.505) | 0.614 |  | 0.138 (-0.266 – 0.541) | 0.477 |  | 0.161 (-0.280 – 0.603) | 0.445 |
| Body fat (%) |  |  |  |  | 0.141 (-0.085 – 0.366) | 0.201 |  | 0.083 (-0.328 – 0.493) | 0.670 |
| VO_2peak_ |  |  |  |  |  |  |  | -0-055 (-0.376 – 0.266) | 0.716 |
| VEGF - maximal | 0.233 (0.054) | 0.027 (0.004 – 0.05) | 0.024 | 0.190 (0.119) | 0.025 (0.000 – 0.050) | 0.051 | 0.187 (0.157) | 0.026 (0.001 – 0.052) | 0.044 |
| Age |  | 0.168 (-0.238 – 0.573) | 0.391 |  | 0.183 (-0.242 – 0.609) | 0.371 |  | 0.111 (-0.348 – 0.569) | 0.611 |
| Body fat (%) |  |  |  |  | 0.054 (-0.202 – 0.310) | 0.660 |  | 0.192 (-0.209 – 0.593) | 0.319 |
| VO_2peak_ |  |  |  |  |  |  |  | -0.055 (-0.166 – 0.438) | 0.716 |
| Myoglobin - basal | 0.235 (0.052) | 0.251 (-0.421 – 0.932) | 0.438 | 0.189 (0.120) | 0.247 (-0.450 – 0.943) | 0.460 | 0.164 (0.182) | 0.313 (-0.423 – 1.048) | 0.376 |
| Age |  | 0.193 (-0.090 – 0.475) | 0.167 |  | 0.187 (-0.107 – 0.481) | 0.193 |  | 0.201 (-0.102 – 0.504) | 0.176 |
| Body fat (%) |  |  |  |  | -0.023 (-0.151 – 0,105) | 0.707 |  | -0.085 (-0.303 – 0.133) | 0.416 |
| VO_2peak_ |  |  |  |  |  |  |  | -0.060 (-0.227 – 0.108) | 0.457 |

|  | Model 1 | | | Model 2 | | | Model 3 | | | | | |  |
| --- | --- | --- | --- | --- | --- | --- | --- | --- | --- | --- | --- | --- | --- |
|  | *Adj. R² (p-value)* | *ß (95% CI)* | *p-value* | *Adj. R² (p-value)* | *ß (95% CI)* | *p-value* | | *Adj. R²*  *(p-value)* | | *ß (95% CI)* | | *p-value* | |
| STAT3 - maximal | **0.415 (0.028)** | **5.994 (1.788 – 10.201)** | **0.010** | **0.473 (0.033)** | **4.716 (0.199 – 9.232)** | **0.042** | | **0.495 (0.047)** | | **4.992 (0.450 – 9.534)** | | **0.035** | |
| Age |  | -0.052 (-0.704 – 0.601) | 0.864 |  | 0.111 (-0.567 – 0.788) | 0.720 | |  | | -0.082 (-0.858 – 0.693) | | 0.812 | |
| Body fat (%) |  |  |  |  | 0.177 (-0.099 – 0.453) | 0.181 | |  | | 0.325 (-0.076 – 0.726) | | 0.098 | |
| VO_2peak_ |  |  |  |  |  |  | |  | | 0.155 (-0.149 – 0.459) | | 0.274 | |
| Glucose - basal | **0.380 (0.011)** | 0.078 (-0.002 – 0.159) | 0.056 | **0.358 (0.028)** | 0.080 (-0.003 – 0.163) | 0.057 | | **0.373 (0.037)** | | **0.092 (0.007 – 0.177)** | | **0.036** | |
| Age |  | 0.194 (-0.013 – 0.400) | 0.064 |  | 0.183 (-0.032 – 0.398) | 0.090 | |  | | 0.215 (-0.006 – 0.436) | | 0.056 | |
| Body fat (%) |  |  |  |  | -0.033 (-0.148– 0.082) | 0.547 | |  | | -0.119(-0.311 – 0.073) | | 0.203 | |
| VO_2peak_ |  |  |  |  |  |  | |  | | -0.081 (-0.226 – 0.064) | | 0.251 | |
| Glucose – maximal | 0.214 (0.065) | 0.192 (-0.022 – 0.362) | 0.029 | 0.227 (0.089) | 0.185 (0.015 –  0.355) | 0.035 | | 0.169 (0.177) | | 0.182 (-0.002 – 0.365) | | 0.052 | |
| Age |  | 0.012 (-0.423 – 0.447) | 0.955 |  | 0.052 (-0.389 – 0.493) | 0.805 | |  | | 0.042 (-0.435 – 0.519 | | 0.852 | |
| Body fat (%) |  |  |  |  | 0.123 (-0.112 – 0.358) | 0.282 | |  | | 0.149 (-0.265 – 0.562) | | 0.452 | |
| VO_2peak_ |  |  |  |  |  |  |  | | 0.024 (-0.289 – 0.337) | | 0.869 | |  |
| HbA1c – maximal | 0.174 (0.093) | -5.736 (- 11,317– -0.156) | 0.045 | **0.310 (0.043)** | **-6.865 (-12.141 –**  **-1.589)** | **0.014** | 0.262 (0.093) | | -6.729 (-12.309 – 1.148) | | 0.022 | |  |
| Age |  | 0.167 (-0.254 – 0.588) | 0.410 |  | 0.224 (-0.168 – 0.616) | 0.214 |  | | 0.202 (-0.233 – 0.638) | | 0.334 | |  |
| Body fat (%) |  |  |  |  | 0.211 (-0.017 – 0.438) | 0.067 |  | | 0.252 (-0.126 – 0.630) | | 0.173 | |  |
| VO_2peak_ |  |  |  |  |  |  |  | | 0.041 (-0.249 – 0.331) | | 0.304 | |  |
